# Preoperative CT-based Intratumoral and Peritumoral Radiomics Prediction for Vasculogenic Mimicry in Lung Adenocarcinoma

**DOI:** 10.2174/0115734056383032250320041531

**Published:** 2025-04-11

**Authors:** Shuhua Li, Yang Li, Ying Meng, Jingcheng Huang, Yihong Gu, Yan Song, Shuni Zhang, Zhiya Zhang, Weiming Zhao, Zongyu Xie

**Affiliations:** 1 Department of Radiology, The First Affiliated Hospital of Bengbu Medical University, Bengbu 233004, China; 2 Department of Medical Imaging Diagnostics, Bengbu Medical University, Bengbu 233030, China; 3 Anhui Province Key Laboratory of Respiratory Tumor and Infectious Disease, Bengbu 233004, China; 4 Department of Radiology, Hospital of Stomatology Affiliated to Anhui Medical University, Hefei 230032, China; 5 Department of Radiology, Jieshou City People’s Hospital, Fuyang 236500, China

**Keywords:** Lung adenocarcinoma, Vasculogenic mimicry, Radiomics, Computed tomography, Prognosis prediction, Radiomics mode

## Abstract

**Objective:**

This study seeks to assess vasculogenic mimicry (VM) occurrence in lung adenocarcinoma (LUAD) by delineating intratumoral and peritumoral characteristics using preoperative CT-based radiomics and a nomogram for enhanced precision.

**Materials and methods:**

Our retrospective analysis enrolled 150 LUAD patients, ascertained their VM status, and stratified them randomly into development (n=105) and validation cohorts. We extracted radiomics features from intra- and peritumoral zones, delineating 3, 5, and 7mm expansions on thin-section chest CT images. We formulated logistic models encompassing a clinical model (CM), intratumoral radiomics model (TRM), peritumoral radiomics models at 3, 5, and 7 mm (PRMs), and a composite model integrating both intra- and peritumoral zones (CRM). A radiomics nomogram model (RNM) was devised, amalgamating the Rad-scores from intra- and peritumoral regions with clinical-radiological traits to forecast VM. The models' efficacy was gauged *via* the receiver operating characteristic (ROC) curve analysis, calibration assessment, and decision curve analysis (DCA).

**Results:**

The CRM outperformed its counterparts, the TRM, PRM_3mm, PRM_5mm, and PRM_7mm models, with AUCs reaching 0.859 and 0.860 in the development and validation cohorts. Within the CM, tumor size and spiculation emerged as significant predictive covariates. The RNM, integrating independent predictors with the CRM-Rad-score, demonstrated clinical utility, achieving AUCs of 0.903 and 0.931 in the respective cohorts.

**Conclusion:**

Our findings underscore the potential of CT-based radiomics characteristics derived from intratumoral and peritumoral regions to assess VM presence in LUAD patients. Combining radiomics signatures with clinicoradiological parameters within a nomogram framework significantly enhances predictive accuracy.

## 1 INTRODUCTION

As the histological majority of non-small cell carcinomas, Lung adenocarcinoma (LUAD), constitutes nearly 40% of pulmonary malignancies while remaining a persistent contributor to global oncological mortality [1]. While emerging multimodal approaches have been developed, five-year survival outcomes in LUAD patients remain disappointingly low at approximately 18% [2, 3]. Neovascularization, essential for tumor growth, proliferation, and metastasis, has been recognized as a key driver of tumor progression over the past decades [4]. Anti-angiogenic therapies, designed to target the tumor microenvironment and disrupt established vasculature, have become a cornerstone of advanced LUAD treatment [5-7]. However, the full therapeutic potential of these treatments has yet to be realized due to the intricate and dynamic nature of the tumor microenvironment [8].

Vasculogenic mimicry (VM) represents a distinct and elusive form of tumor microcirculation, in which cancer cells autonomously form blood vessel-like networks without endothelial cell participation [9]. This process is frequently associated with aggressive malignancies and is a predictor of adverse clinical outcomes [10, 11]. The presence of VM significantly impedes the efficacy of anti-angiogenic drugs [12], underscoring the urgent need for novel therapeutic targets and prognostic biomarkers in LUAD.

Radiomics, leveraging a noninvasive methodology to quantify a vast array of features from medical imaging, presents a compelling strategy for biomarker identification. By converting images into high-dimensional datasets, this approach elucidates the morphological nuances and tumor heterogeneity [13], and enhances the detection of subtle imaging features associated with VM. Early research has shown radiomics' efficacy in forecasting critical LUAD biomarkers, including EGFR mutation status [14], spread through air space (STAS) [15], and PD-L1 expression [16]. Previous radiomics studies have primarily concentrated on other LUAD biomarkers or clinical outcomes. However, the application of CT-based radiomics for VM status prediction in LUAD remains underexplored, signifying a notable area for future investigation.

Given VM's role as an invasive phenotype, it may present in the intra- and peritumoral regions, potentially impacting the clinical course of the tumor. Although radiomics has garnered considerable attention, the application of intra- and peritumoral radiomics biomarkers for VM detection is sparse, reflecting a notable research gap. The current study introduces an innovative nomogram that integrates radiomics data from intratumoral and peritumoral zones with established clinical parameters to address this. By doing so, we aim to provide a comprehensive and nuanced approach to VM assessment, thereby advancing personalized and optimized therapeutic strategies for LUAD patients.

## 2 MATERIALS AND METHODS

### 2.1 Patients

From January 2019 to December 2021, 320 LUAD patients who underwent the non-enhanced chest CT scan and were pathologically confirmed in the First Affiliated Hospital of Bengbu Medical University were assessed retrospectively. Inclusion criteria comprised: (a) pathologically confirmed as LUAD with complete clinicopathological data; (b) non-enhanced chest CT conducted within 2 weeks pre-surgery; (c) VM status determined *via* immunohistochemical staining; (d) no history of other malignancies or prior treatments affecting the lung lesions. Exclusion criteria are systematically outlined below: (a) suboptimal imaging quality or artifacts (n=15); (b) unavailability of thin-slice CT images (n=78); (c) lesions too small for clear delineation (n=24); (d) presence of multiple lung lesions (n=19); (e) with the presence of underlying lung pathology (n=34).

After applying these criteria, 150 patients were enrolled, comprising 47 with VM-positive status and 103 with VM-negative status. The study group included 92 females and 58 males, with a mean age of 58.92 ± 10.77 years (range: 29 - 87). The participants were randomly allocated into a development cohort of 105 and a validation cohort of 45 in a 7:3 ratio. As shown in Fig. (**1**).

### 2.2 Histopathological Evaluation

The histopathological evaluation employed PAS (BA4080B; Baso)/CD34 (Kit-0004; MXB) co-staining to visualize VM patterns through glycosylated basement membrane localization. VM identification required the concurrent presence of CD34−/PAS+ neoplastic cells with erythrocyte-containing, tumor-encased luminal structures, per established criteria [10, 17].

### 2.3 CT Acquisition and Characteristic Analysis

CT scans were conducted with subjects in a supine position, arms elevated post-deep inspiration. Unenhanced CT images were collected using two state-of-the-art CT scanners: Revolution Apex 256S (GE Healthcare) and Light Speed VCT 64S (GE Healthcare). Scanning parameters were configured with a tube voltage of 120 kVp, and an automatic exposure control system to modulate the tube current. The imaging field was configured to 400×400 mm with a 512×512pixel matrix density. CT acquisitions employed 5-mm collimation (thickness/interval) with 0.625-mm isotropic reconstruction.

CT interpretations were independently performed by dual-blinded thoracic radiologists (R1:7-yr/R2:18-yr experience), with consensus arbitration by senior consultant R3 (25-yr). The CT characteristics analyzed were extensive and included: nodule size, boundary clarity, shape regularity, presence of lobulation and spiculation signs, density categorization, halo sign, calcification, necrosis, vacuole sign, cavity presence, air bronchogram, peripheral fibrosis, pleural retraction, and lymph node size (< 10 mm or ≥ 10 mm).

### 2.4 Image Segmentation

All tumor segmentation with lung (1500/-600 HU) window setting was executed using ITK-SNAP (version 3.8.0). Following a consensus by radiologists R1 and R2, manual segmentation was performed meticulously. Regions of interest (ROIs) were delineated along the tumor margins across axial, sagittal, and coronal views, employing a systematic, layer-by-layer methodology. Throughout this process, there was a deliberate effort to exclude bronchi, blood vessels, and pleura from the ROIs, ensuring that the focus remained on the tumor tissue [18]. This thorough segmentation process created a three-dimensional volume of interest (VOI), an integrated assembly of all two-dimensional ROIs.

A previous study [19] quantified the extent of lung cancer microinvasion *via* histopathological confirmation and demonstrated distinct adenocarcinoma (2.69mm) versus squamous cell carcinoma (1.48mm) microscopic extension margins. Tumors exhibiting “vascular” invasion or spiculated margins demonstrated greater microscopic spread than those lacking these characteristics. Additionally, radiomics analysis within a 5 mm peritumoral zone has unveiled tumor heterogeneity in non-small cell lung cancer [20]. Further studies have indicated 3-9mm peri-tumor zones as critical biomarkers for deciphering LUAD intratumoral heterogeneity [21].

Informed by these findings, volumetric expansion (3/5/7mm) transformed tumor volume of interest (VOI-T) into standardized peritumoral VOI (VOI-P) through automated morphological dilation, namely VOI-P3, VOI-P5, and VOI-P7.

### 2.5 Radiomics Feature Extraction

Each VOI underwent standardized radiomics phenotyping (PyRadiomics v3.2.0, https:// pyradiomics. readthedocs. io/) quantifying 1,781 biomarkers across morphological/ textural/ statistical domains. These included first-order statistical attributes, morphological descriptors in both 3D and 2D domains, and a suite of textural features derived from Co-occurrence (GLCM), Run-Length (GLRLM), Size-Zone (GLSZM), Tone-Difference (NGTDM), and Dependence (GLDM) matrices. To mitigate the impact of feature scaling on the subsequent selection process, we implemented a rigorous normalization protocol. Each radiomics feature was scaled linearly to a uniform range of [-1, 1], employing a maximum absolute value normalization technique as detailed in Appendix S1. Ensuring methodological robustness, we appraised the concordance of feature extraction between two radiologists. Only those features demonstrating an intra-class correlation coefficient (ICC) exceeding 0.75 were advanced to the subsequent screening phase, signifying high reliability. The feature selection process was further refined through an optimal screening strategy, where we identified features with significant discriminatory power for classification. Feature selection was executed through two-phase analytics:​ univariate screening​with ANOVA-based metrics (f_classif implementation in Darwin v3.3.8) in Appendix S2; ​multivariate refinement​via LASSO-λ regularization (10-fold cross-validated coefficients).

### 2.6 Model Development and Validation

Synthetic minority over-sampling technique (SMOTE)-based resampling was employed to mitigate dataset imbalance. We employed the Support Vector Machine (SVM) algorithm to develop the intratumoral radiomics model (TRM) and the peritumoral radiomics models (PRMs) at intervals of 3/5/7 mm, respectively. Based on the radiomics features with the highest contribution, corresponding Rad-scores were computed for each model to quantify their predictive power. Independent clinical-radiological predictors were identified to establish the clinical model (CM), a foundation for our predictive framework. To construct a combined intra- and peritumoral radiomics model (CRM), the peritumoral region exhibiting optimal discriminatory performance based on the area under the receiver operating characteristic curve (AUC) was selected. Subsequently, we crafted a comprehensive radiomics nomogram model (RNM) by integrating the intra-tumoral and peritumoral Rad-scores alongside the clinical-radiological characteristics. A 10-fold cross-validation framework was employed to systematically assess model generalizability and performance stability across diverse data partitions. The entire workflow, meticulously designed to enhance clarity and reproducibility, is vividly depicted in Fig. (**2**).

### 2.7 Statistical Analysis

Data analysis was implemented in SPSS 23.0, MedCalc 19.1.2, and R 4.2.2. Continuous variables underwent normality evaluation *via* the Kolmogorov-Smirnov test, whereas categorical variables were analyzed with chi-square or Fisher's exact test. Distribution-dependent comparisons of continuous data utilized the independent t-test or Mann-Whitney U test. Model stability was evaluated through tenfold cross-validation. Independent clinical-radiological predictors were extracted from both univariate and multivariate logistic regression. The radiomics nomogram was constructed using R software. For model comparison, receiver operating characteristic (ROC) curves were analyzed to derive AUC values, sensitivity, and specificity across VOIs, with statistical differences assessed *via* the Delong test. Model consistency and clinical utility were further evaluated using calibration and decision curve analysis (DCA). A threshold of *P* < 0.05 defined statistical significance.

## 3 RESULTS

### 3.1 Baseline Clinical-radiological Profiles of LUAD Cohorts

Table **1** summarizes the clinical-radiological features of LUAD patients in the development and validation cohorts. The two groups exhibited comparable distributions across all parameters (*P* > 0.05), confirming effective cohort matching.

Within the development cohort, tumors demonstrating VM+ displayed distinct clinical-radiological profiles compared to VM- counterparts, particularly in terms of size, boundary, spiculation sign, density, halo sign, and pleural retraction (all *P* < 0.01, except for halo sign *P* = 0.03 and pleural retraction *P* = 0.049). Similarly, in the validation cohort, where VM+ tumors demonstrated additional divergent features, including size, spiculation, pleural retraction (all *P* < 0.01), and vacuole sign (*P* = 0.02) compared to VM- tumors.

### 3.2 Radiomics Feature Extraction and Radiomics Signature Construction

Following feature selection *via* LASSO regression analysis, 6, 4, 5, and 4 optimal radiomics features (Table **S1**) were identified from VOI-T, VOI-P3, VOI-P5, and VOI-P7, respectively, based on nonzero coefficients from the SVM. The rad-score was computed as the weighted sum of selected features plus a constant (Appendix **S3**). As detailed in Table **2**, the TRM’s AUCs achieved values of 0.821 (95% confidence interval (CI), 0.734 - 0.889) and 0.847 (95% CI, 0.708 - 0.937) in the development and validation cohorts. In the development cohort, all peritumoral models (PRM_3 mm, PRM_5 mm, PRM_7 mm) demonstrated robust performance, yielding AUCs of 0.798, 0.804, and 0.756, while the validation cohort exhibited corresponding AUCs of 0.824, 0.833, and 0.791 (Table **2** and Fig. **S1**). Among these, the 5 mm peritumoral region (PRM_5) emerged as the most predictive, displaying the highest AUC values (0.804 and 0.833) in both cohorts. To enhance predictive capacity, the TRM radiomics signature was integrated with PRM_5 to form the combined radiomics model (CRM), from which 6 optimal features were ultimately retained (Table **S1**).

Rad-Score = -1.084*lbp-3D-k_glszm_ZonePercentage_HRCT_paramsName4-0.808*log-sigma-3-0-mm-3D_firstorder_Maximum_HRCT_paramsName4-0.761*log-sigma-3-0-mm-3D_firstorder_Median_HRCT_paramsName4-0.666*logarithm_ngtdm_Contrast_HRCT_paramsName4-0.559*log-sigma-3-0-mm-3D_glszm_SizeZoneNonUniformity_HRCT_paramsName4-0.399*logarithm_firstorder_RobustMeanAbsoluteDeviation_HRCT_paramsName4+2.102. K-means clustering with k set to 2 was utilized to visualize multicollinearity among the selected features. This analysis of the optimal radiomics features from VOI-T, VOI-P3, VOI-P5, VOI-P7, and the combined VOI-T+P5 demonstrated significant associations between radiomics profiles and VM status (Fig. **S2**).

### 3.3 Model Development and Validation

**Fig. (3):**
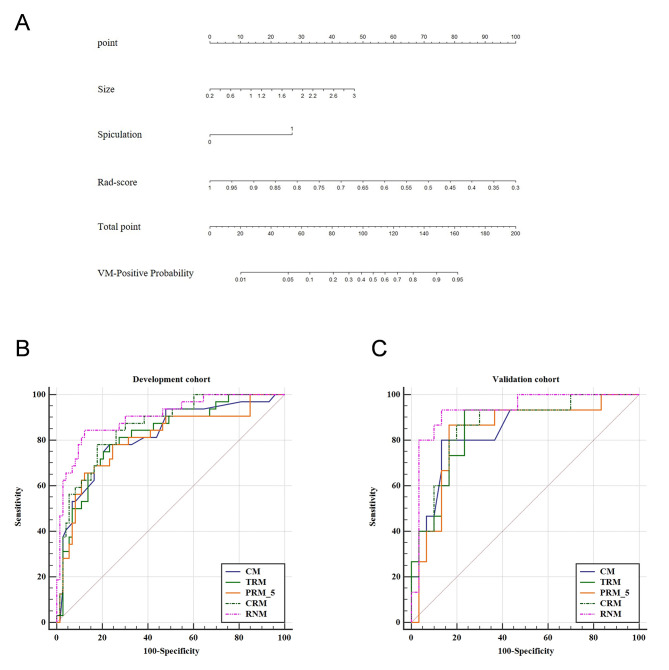
The radiomics nomogram was developed based on the size, spiculation, and Rad-score of VOI-T+P5 (**A**). ROC curves of the development cohort (**B**) and validation cohort (**C**). CM, clinical model; CRM, combined intra- and peritumoral radiomics model; VM, vasculogenic mimicry; PRM_5, 5-mm peritumoral radiomics model; RNM, radiomics nomogram model; TRM, tumoral radiomics model.

**Fig. (4):**
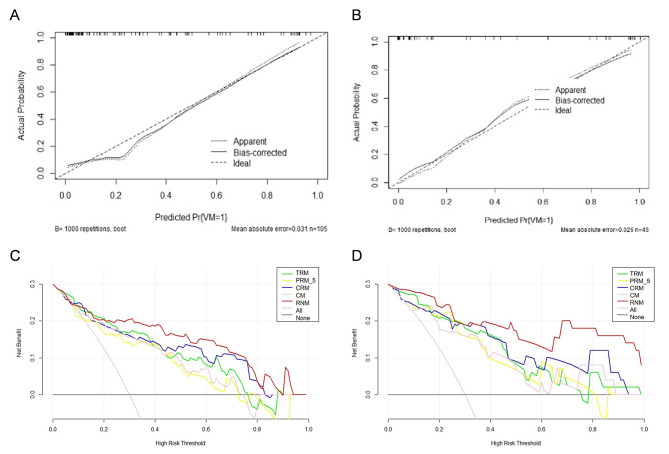
Calibration curves of the development cohort (**A**) and validation cohort (**B**). The decision curve (DCA) of CM, TRM, PRM_5, CRM, and RNM in the development cohort (**C**) and validation cohort (**D**). RNM remains superior in most threshold ranges in two cohorts. CM, clinical model; CRM, combined intra- and peritumoral radiomics model; PRM_5, 5-mm peritumoral radiomics model; RNM, radiomics nomogram model; TRM, tumoral radiomics model.

In the development cohort, spiculation sign (95% CI: 0.040 - 0.428; P = 0.001) and tumor size (95% CI: 1.505 - 8.640; P = 0.004) were identified as significant predictors of LUAD with VM positivity, as detailed in Table **S2**. The CM, incorporating these factors, demonstrated AUCs of 0.819 (95% CI: 0.732 - 0.887) in the development cohort and 0.834 (95% CI: 0.694 - 0.928) in the validation cohort. Comparatively, the TRM yielded AUCs of 0.821 (95% CI: 0.734-0.889) and 0.847 (95% CI: 0.708-0.937) in the two cohorts. The CRM exhibited enhanced discriminatory capability with AUCs of 0.859 (95% CI: 0.777 - 0.919) and 0.860 (95% CI: 0.724 - 0.945) in the respective cohorts (Table **2** and Fig. **3A, B**). RNM outperformed all other models, achieving AUCs of 0.903 (95% CI: 0.829-0.952) and 0.931 (95% CI: 0.814-0.985) in the respective cohorts (Table **2** and Fig. **3**). Notably, the incremental AUC improvements from CM to RNM (ΔAUC: +0.084 in the development cohort) suggest that integrating additional biomarkers or radiomics features may refine risk stratification. All models, including TRM, PRM_3, PRM_5, PRM_7, CRM, and RNM, exhibited excellent stability following 10-fold cross-validation, as documented in Fig. (**S3**). The DeLong test revealed significant differences in AUC between RNM and TRM (P = 0.042), PRM_5 (P = 0.020), and CRM (P = 0.048) in the development cohort, though no such differences were observed in the validation cohort (P > 0.05 for all comparisons; Table **3**). This discrepancy may reflect reduced statistical power in the smaller validation cohort of RNM in the development phase, warranting external validation in larger populations. Calibration curves confirmed strong agreement between RNM-predicted and observed VM probabilities in both cohorts (Fig. **4A** and **B**). Furthermore, DCA demonstrated that the RNM provided superior net benefits over other models for VM status prediction in both cohorts (Fig. **4C** and **D**).

## 4 DISCUSSION

Expression data and clinical correlates of genes associated with VM suggested that VM could serve as an essential biomarker for assessing lung adenocarcinoma's prognosis and immune context, potentially representing a novel target for therapeutic intervention in oncology [22]. Given the presence of VM, surgeons may perform more extensive tumor resections to reduce residual cells and VM structure formation. Postoperative adjuvant therapies such as chemotherapy, radiotherapy, and targeted treatments may be adjusted based on VM expression to enhance effectiveness.

This study assessed the predictive capacity of intratumoral, peritumoral, and integrated clinical-radiological characteristics for VM status in LUAD using radiomics models. The RNM, integrating multi-region radiomics biomarkers with clinicoradiological variables, outperformed CRM and CM in predicting VM status for LUAD patients. The integration of RNM into clinical workflows may facilitate personalized treatment plans, particularly in guiding adjuvant therapy decisions for VM-positive LUAD patients who face higher risks of metastasis and recurrence.

Our findings suggested that integrating radiomics features from both intra- and peritumoral regions of LUAD enhances predictive accuracy for VM status, as evidenced by improved AUC values. This aligns with prior studies demonstrating intratumoral and peritumoral area values for predicting prognostic biomarkers in malignant tumors [14, 23, 24]. Our study represents the first to explore the predictive capacity of intra- and peritumoral radiomics features for VM expression in LUAD. This pioneering approach has yet to be previously documented, highlighting the novelty of our findings in the field of LUAD research. By integrating clinical-radiological characteristics with radiomics features from both regions, we have developed a robust predictive model for VM status in LUAD patients. This innovative application of radiomics and clinical data fusion offers a significant advancement in the prediction of VM, which is a critical factor in LUAD prognosis and treatment response, marking a significant expansion in the scope of radiomics for prognostic biomarker prediction in malignant tumors.

A prior study [25] explored radiomics methods for predicting VM status in LUAD, but its scope was restricted to intratumoral characteristics, omitting data from peritumoral regions. In contrast, our work adopted a dual-region strategy, integrating both intra- and peritumoral radiomics features to evaluate their synergistic utility in predicting VM status. This methodology aligns with the understanding that invasive tumor cells—capable of structural disruption and metastatic spread—alter adjacent parenchymal morphology and texture [26]. Tumor heterogeneity extends beyond the tumor, encompassing the microenvironment, which consists of diverse cellular and acellular elements [27, 28]. While the microscopic environment around a lesion may not be readily apparent on medical imaging, radiomics features of the peritumoral region can quantify its heterogeneity, revealing subtleties beyond the naked eye's resolution [29, 30]. Highly aggressive tumor cells can sustain their metabolic demands through deformation and extracellular matrix remodeling to form vasculogenic channels known as VM. VM is an endothelium-independent tumor microcirculation mode that promotes tumor cells to secrete proteolytic enzymes and degrade the basement membrane and extracellular matrix. This process enhances blood perfusion, thereby driving tumor progression and metastatic dissemination [31]. Several mechanisms within the tumor microenvironment contribute to VM generation, including tumor stem cells, cancer-associated fibroblasts (CAFs), epithelial-mesenchymal transition (EMT), and tumor-associated macrophages (TAMs) [32]. LUAD exhibits pronounced cellular and mutational heterogeneity, and VM within the tumor microenvironment presents a potential target for lung cancer therapy [22]. Consequently, the expression of VM in LUAD is related to intratumoral and peritumoral microenvironment changes; these may serve as the pathophysiological foundation for the superior predictive efficacy of integrated models compared to TRM or PRM.

Our analysis systematically evaluated radiomics features derived from VOI-T and VOI-P5 regions, ultimately selecting 6, 5, and 6 features from VOI-T, VOI-P5, and the combined VOI-T + P5, respectively. These features encompassed first-order statistics, GLCM, NGTDM, and GLSZM descriptors, demonstrating good stability and reproducibility across regions, suggesting minimal influence from the delineation of regions [33]. First-order statistics features capture the symmetry, uniformity, and localized intensity distribution of tumor voxels, serve as radiographic indicators of tumor heterogeneity, reflecting elevated malignant potential [34]. Extracted texture features from tumor and peritumoral regions enable holistic assessment of spatial and directional heterogeneity across multiscale contexts [35]. Texture-based biomarkers are grounded in their capacity to noninvasively capture intrinsic biological aggressiveness, such as hypoxia, proliferation, angiogenesis, necrosis, etc., that predict adverse reactions to treatment and prognosis [36]. This heterogeneity can be reflected in pretreatment CT images. Our findings indicated that CRM and RNM that took CM and CRM into account exhibited enhanced predictive accuracy for VM status in LUAD, achieving AUC values of 0.859 and 0.903, indicating that VM-positive LUAD has a coarser texture and more significant tumor heterogeneity in intratumoral and peritumoral regions.

Our analysis of clinical-radiological factors revealed size and spiculation as significant predictors of VM status in LUAD. This is congruent with prior studies correlating size and spiculation with VM status [25, 37]. A possible explanation is that as the tumor grows, it is more likely to cause tumor hypoxia, which contributes to VM formation by inducing EMT [38]. It may be a potential reason for size being related to VM. This result suggests that VM could facilitate LUAD tumor growth. A possible reason spiculation is associated with VM status is that the tumor with spiculation has abundant CAFs derived from epithelial cells through EMT [39]. In the tumor microenvironment, EMT and CAFs promote VM formation [32, 40]. To optimize predictive accuracy, clinical-radiological variables were integrated into an RNM combining intratumoral and peritumoral features. This model exhibited robust discriminative power for VM prediction, with decision curve analysis (DCA) further validating its clinical utility.

Our study has several limitations that warrant acknowledgment. First, the retrospective single-institution design may introduce inherent selection bias. Prospective multi-institutional cohorts are required to verify the generalizability and reproducibility of the model. Second, only intratumoral and peritumoral radiomics features were evaluated in the study. Future efforts will incorporate automated deep-learning frameworks to enhance feature extraction. Third, while spatial heterogeneity within tumors was partially addressed, a more granular subregional radiomics analysis is needed. Subsequent research will investigate the utility of subcompartmental radiomics in predicting VM expression in LUAD.

## CONCLUSION

In summary, radiomics signatures derived from intra- and peritumoral regions in LUAD offer complementary insights for identifying VM. The nomogram developed in this study, integrating CT-based radiomics features from both tumor regions with clinicoradiological variables, demonstrated robust predictive capability for assessing VM expression in LUAD.

## Figures and Tables

**Fig. (1):**
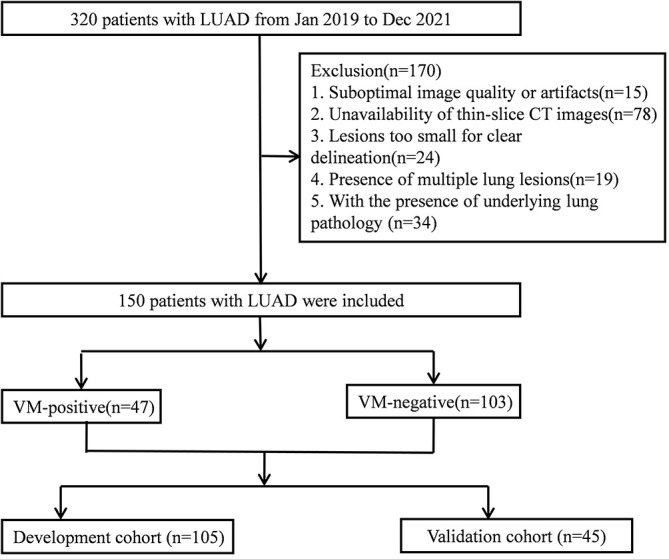
Patient recruitment pathway in this study. Note: LUAD, Lung adenocarcinoma; VM, vasculogenic mimicry.

**Fig. (2):**
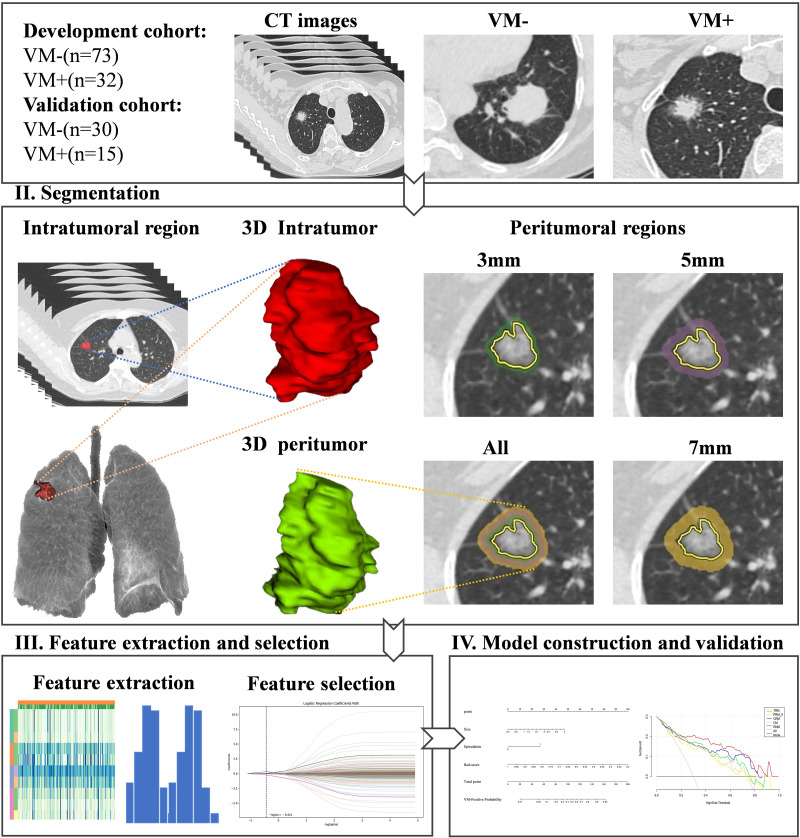
Workflow of the study. VM-, vasculogenic mimicry negative; VM+, vasculogenic mimicry positive. Panel II illustrates the regions within and around a tumor from a patient's case. The diagram depicts the intra-tumoral area and the peritumoral regions at distances of 3mm, 5mm, and 7mm from the tumor margin.

**Table 1:** Clinical and radiological baseline characteristics of LUAD in the development and validation cohorts.

Characteristics	Development Cohort (N=105)					Validation Cohort (N=45)					*P*
	All	VM(+)	VM(-)	*t/Z/χ^2^*Value	*P*	All	VM(+)	VM(-)	*t/Z/χ^2^*Value	*P*	
Age	58.78 ± 11.57	60.56 ± 9.55	58.00 ± 12,34	-1.15	0.25	59.24 ± 8.72	58.93 ± 10.47	59.40 ± 7.90	0.15	0.88	0.810
Sex				3.10	0.08				1.57	0.21	0.214
Male	44 (41.9)	20 (62.50)	32 (43.84)			14 (31.1)	7 (46.67)	7 (23.33)			
Female	61 (58.1)	12 (37.50)	41 (56.16)			31 (68.9)	8 (53.33)	23 (76.67)			
Size	1.52 ± 0.75	2.05 (1.50, 22.78)	1.10 (0.80, 1.50)	-5.07	< 0.01	1.50 ± 0.75	2.50 (1.50, 2.70)	1.00 (0.88, 1.50)	- 3.58	< 0.01	0.913
Shape, n (%)				3.60	0.06				0.01	0.91^*^	
Regular	37 (35.2)	7 (21.88)	30 (41.10)			14 (31.1)	4 (26.67)	10 (33.33)			0.625
Irregular	68 (64.8)	25 (78.3)	43 (58.90)			31 (68.9)	11 (73.33)	20 (66.67)			
Boundary, n (%)				8.28	< 0.01				2.19	0.14^*^	0.938
Clear	73 (69.5)	16 (50.00)	57 (78.08)			31 (68.9)	13 (86.67)	18 (60.00)			
Blur	32 (30.5)	16 (50.00)	16 (21.92)			14 (31.1)	2 (13.33)	12 (40.00)			
Lobulation, n (%)				2.80	0.09				3.91	0.05^*^	0.699
Absence	24 (22.9)	4 (12.50)	20 (5.4)			9 (20.0)	0 (0.00)	9 (30.00)			
Present	81(77.1)	28 (87.50)	53 (94.6)			36 (80.0)	15 (100.00)	21 (70.00)			
Spiculation, n (%)				30.02	< 0.01				9.72	< 0.01	0.146
Absence	67 (63.8)	8 (25.00)	59 (80.82)			23 (51.1)	1 (6.67)	20 (66.67)			
Present	38 (36.2)	24 (75.00)	14 (19.18)			22 (48.9)	14 (93.33)	10 (33.33)			
Density, n (%)				9.44	< 0.01				0.71	0.40	0.511
GGO or mGGO	50 (47.6)	8 (25.00)	42 (57.53)			22 (48.9)	6 (40.00)	16 (53.33)			
Consolidation	55 (52.4)	24 (75.00)	31 (42.47)			23 (51.1)	9 (60.00)	14 (46.67)			
Halo sign, n (%)				4.94	0.03				0.72	0.40	0.373
Absence	55 (52.4)	22 (68.75)	33 (45.21)			20 (44.4)	8 (53.33)	12 (40.00)			
Present	50 (47.6)	10 (32.25)	40 (54.79)			25 (55.6)	7 (46.67)	18 (60.00			
Calcification, n (%)				3.87	0.05^*^				0.00	1.00^*^	0.932
Absence	100 (95.2)	28 (87.50)	72 (98.63)			43 (95.6)	14 (93.33)	29 (96.67)			
Present	5 (4.8)	4 (12.50)	1 (1.37)			2 (4.4)	1 (6.67)	1 (3.33)			
Necrosis, n (%)				0.39	0.54^*^				0.00	1.00^*^	0.747
Absence	99 (94.3)	29 (90.62)	70 (95.89)			43 (95.6)	14 (93.33)	29 (96.67)			
Present	6 (5.7)	4 (12.50)	3 (4.11)			2 (4.4)	1 (6.67)	1 (3.33)			
Vocule sign, n (%)				1.99	0.16				5.49	0.02^*^	0.656
Absence	83 (79.0)	28 (87.50)	55 (75.34)			37 (82.2)	9 (60.00)	28 (93.33)			
Present	22 (21.0)	4 (12.50	18 (24.66)			8 (17.8)	6 (40.00)	2 (6.67)			
Cavity, n (%)				0.09	0.76^*^				0.00	1.00^*^	0.321
Absence	99 (94.3)	31 (96.87)	68 (93.15)			44 (97.8)	15 (100.00)	29 (96.67)			
Present	6 (5.7)	1 (3.13)	5 (6.85)			1(2.2)	0 (0.00)	1 (3.33)			
Air bronchogram, n (%)				1.607	0.205				0.045	0.832	0.099
Absence	73 (69.5)	25 (78.1)	48 (65.8)			25 (55.6)	8 (53.3)	17 (56.7)			
Present	32 (30.5)	7 (21.9)	25 (34.2)			20 (44.4)	7 (46.7)	13 (43.3)			
Peripheral fibrosis, n (%)				2.95	0.09				0.16	0.69^*^	0.895
Absence	83 (79.0)	22 (68.75)	61 (83.56)			36 (80.0)	11 (73.33)	25 (83.33)			
Present	22 (21.0)	10 (31.25)	12 (16.44)			9 (20.0)	4 (26.67)	5 (16.67)			
Pleural retraction, n (%)				3.88	0.049				5.45	0.02	0.886
Absence	48 (45.7)	10 (31.25)	38 (52.05)			20 (44.4)	3 (20.00)	17(56.67)			
Present	57 (54.3)	22 (68.75)	35 (47.95)			25 (55.6)	12 (80.00)	13(43.33)			
Lymphadenectasis, n (%)				0.00	1.00^*^				0.00	1.00^*^	0.836
< 10mm	97 (92.4)	30 (93.75)	67 (91.78)			42 (93.3)	14 (93.33)	28 (93.33)			
≥ 10mm	8 (7.6)	2 (6.25)	6 (8.22)			3 (6.7)	1 (6.67)	2 (6.67)			

Note: *Continuous correction χ2 test. GGO, ground-glass opacity; LUAD, lung adenocarcinoma; mGGO, mixed ground-glass opacity; VM, vasculogenic mimicry. *P *< 0.05 is considered statistically significant.

**Table 2:** Prediction performance of the models in development and validation cohorts.

Cohort	Model	AUC (95% CI)	Accuracy	Sensitivity	Specificity
Development Cohort	CM	0.819 (0.732 to 0.887)	0.771	0.781	0.767
	TRM	0.821 (0.734 to 0.889)	0.771	0.781	0.767
	PRM_3	0.798 (0.708 to 0.870)	0.774	0.750	0.795
	PRM_5	0.804 (0.715 to 0.875)	0.772	0.781	0.753
	PRM_7	0.756 (0.662 to 0.835)	0.763	0.781	0.699
	CRM	0.859 (0.777 to 0.919)	0.779	0.781	0.822
	RNM	0.903 (0.829 to 0.952)	0.895	0.800	0.867
Validation Cohort	CM	0.834 (0.694 to 0.928)	0.851	0.800	0.867
	TRM	0.847 (0.708 to 0.937)	0.882	0.933	0.767
	PRM_3	0.824 (0.682 to 0.922)	0.851	0.867	0.733
	PRM_5	0.833 (0.692 to 0.928)	0.862	0.867	0.833
	PRM_7	0.791 (0.644 to 0.898)	0.822	0.800	0.733
	CRM	0.860 (0.724 to 0.945)	0.851	0.867	0.800
	RNM	0.931 (0.814 to 0.985)	0.902	0.933	0.867

**Note: **AUC, area under the curve; CI, confidence interval; CM, clinical model; CRM, combined intra- and peritumoral-5mm radiomics model; PRM_3, 3-mm peritumoral radiomics model; PRM_5, 5-mm peritumoral radiomics model; PRM_7, 7-mm peritumoral radiomics model; RNM, radiomics nomogram model; TRM, intratumoral radiomics model.

**Table 3:** Comparison of ROC curves in the development and validation cohorts.

Development Cohort			Validation Cohort		
Compares	Z Statistic	*P*	Compares	Z Statistic	*P*
CM *vs* TRM	0.0331	0.9736	CM *vs* TRM	0.117	0.9072
CM *vs* PRM_5	0.263	0.7926	CM *vs* PRM_5	0.0110	0.9913
CM *vs* CRM	0.783	0.4338	CM *vs* CRM	0.304	0.7613
CM *vs* RNM	2.518	0.0118	CM *vs* RNM	1.651	0.0987
TRM *vs* PRM_5	0.423	0.6726	TRM *vs* PRM_5	0.179	0.8582
TRM *vs* CRM	1.026	0.3050	TRM *vs* CRM	0.168	0.8670
TRM *vs* RNM	2.034	0.0420	TRM *vs* RNM	1.080	0.2803
PRM_5 *vs* CRM	1.260	0.2076	PRM_5 *vs* CRM	0.360	0.7186
PRM_5 *vs* RNM	2.335	0.0196	PRM_5 *vs* RNM	1.302	0.1929
CRM *vs* RNM	1.981	0.0476	CRM *vs* RNM	1.928	0.0538

**Note: **CM, clinical model; CRM, combined intra- and peritumoral-5mm radiomics model; ROC, receiver operating characteristic; PRM_5, 5-mm peritumoral radiomics model; RNM, radiomics nomogram model; TRM, intratumoral radiomics model. *P* is derived from Delong test between each of the ROCs, and *P* < 0.05 is considered statistically significant.

## Data Availability

The data supporting this study's findings are available upon reasonable request from the corresponding author [Z.X].

## LIST OF ABBREVIATIONS

AUC: Receiver operating characteristics curve
CI: Confidence interval
LUAD: Lung adenocarcinoma
CM: Clinical model
CRM: Combined intra- and peritumoral radiomics model
DCA: Decision curve analysis
LASSO: Least absolute shrinkage and selection operator
VM: Vasculogenic mimicry
PRM: Peritumoral radiomics model
RNM: Radiomics nomogram model
ROC: Receiver operating characteristic
ROI: Region of interest
TRM: Intratumoral radiomics model
VOI: Volume of interest

## STANDARDS OF REPORTING

STROBE guidelines were followed.

## References

[1] Denisenko T.V., Budkevich I.N., Zhivotovsky B. (2018). Cell death-based treatment of lung adenocarcinoma.. Cell Death Dis..

[2] Hirsch F.R., Scagliotti G.V., Mulshine J.L., Kwon R., Curran W.J., Wu Y.L., Paz-Ares L. (2017). Lung cancer: Current therapies and new targeted treatments.. Lancet.

[3] Gettinger S., Horn L., Jackman D., Spigel D., Antonia S., Hellmann M., Powderly J., Heist R., Sequist L.V., Smith D.C., Leming P., Geese W.J., Yoon D., Li A., Brahmer J. (2018). Five-year follow-up of nivolumab in previously treated advanced non–small-cell lung cancer: Results from the CA209-003 study.. J. Clin. Oncol..

[4] Lugano R., Ramachandran M., Dimberg A. (2020). Tumor angiogenesis: Causes, consequences, challenges and opportunities.. Cell. Mol. Life Sci..

[5] Tian W., Cao C., Shu L., Wu F. (2020). Anti-angiogenic therapy in the treatment of non-small cell lung cancer.. OncoTargets Ther..

[6] Tan A.C., Pavlakis N. (2022). Anti-angiogenic therapy in ALK rearranged non-small cell lung cancer (NSCLC).. Int. J. Mol. Sci..

[7] Fang H., Sun Q., Zhou J., Zhang H., Song Q., Zhang H., Yu G., Guo Y., Huang C., Mou Y., Jia C., Song Y., Liu A., Song K., Lu C., Tian R., Wei S., Yang D., Chen Y., Li T., Wang K., Yu Y., Lv Y., Mo K., Sun P., Yu X., Song X. (2023). m^6^A methylation reader IGF2BP2 activates endothelial cells to promote angiogenesis and metastasis of lung adenocarcinoma.. Mol. Cancer.

[8] Yan X., Zhao Z., Tang H. (2023). Current status and future of anti-angiogenic drugs in lung cancer.. Clin. Exp. Med..

[9] Simizu S. (2022). Vasculogenic mimicry: A dynamic event of malignancy.. Biochim. Biophys. Acta, Gen. Subj..

[10] He X., You J., Ding H., Zhang Z., Cui L., Shen X., Bian X., Liu Y., Chen J. (2021). Vasculogenic mimicry, a negative indicator for progression free survival of lung adenocarcinoma irrespective of first line treatment and epithelial growth factor receptor mutation status.. BMC Cancer.

[11] Zheng N., Zhang S., Wu W., Zhang N., Wang J. (2021). Regulatory mechanisms and therapeutic targeting of vasculogenic mimicry in hepatocellular carcinoma.. Pharmacol. Res..

[12] Li W., Wu J., Jia Q., Shi Y., Li F., Zhang L., Shi F., Wang X., Wu S. (2024). PD-L1 knockdown suppresses vasculogenic mimicry of non-small cell lung cancer by modulating ZEB1-triggered EMT.. BMC Cancer.

[13] Gillies R.J., Kinahan P.E., Hricak H. (2016). Radiomics: Images are more than pictures, they are data.. Radiology.

[14] Shang Y., Chen W., Li G., Huang Y., Wang Y., Kui X., Li M., Zheng H., Zhao W., Liu J. (2023). Computed Tomography-derived intratumoral and peritumoral radiomics in predicting EGFR mutation in lung adenocarcinoma.. Radiol. Med..

[15] Wang Y., Ding Y., Liu X., Li X., Jia X., Li J., Zhang H., Song Z., Xu M., Ren J., Sun D. (2023). Preoperative CT-based radiomics combined with tumour spread through air spaces can accurately predict early recurrence of stage I lung adenocarcinoma: A multicentre retrospective cohort study.. Cancer Imaging.

[16] Meißner A.K., Gutsche R., Galldiks N., Kocher M., Jünger S.T., Eich M.L., Nogova L., Araceli T., Schmidt N.O., Ruge M.I., Goldbrunner R., Proescholdt M., Grau S., Lohmann P. (2023). Radiomics for the non-invasive prediction of PD-L1 expression in patients with brain metastases secondary to non-small cell lung cancer.. J. Neurooncol..

[17] Wei X., Chen Y., Jiang X., Peng M., Liu Y., Mo Y., Ren D., Hua Y., Yu B., Zhou Y., Liao Q., Wang H., Xiang B., Zhou M., Li X., Li G., Li Y., Xiong W., Zeng Z. (2021). Mechanisms of vasculogenic mimicry in hypoxic tumor microenvironments.. Mol. Cancer.

[18] Chen Q., Shao J., Xue T., Peng H., Li M., Duan S., Feng F. (2022). Intratumoral and peritumoral radiomics nomograms for the preoperative prediction of lymphovascular invasion and overall survival in non-small cell lung cancer.. Eur. Radiol..

[19] Giraud P., Antoine M., Larrouy A., Milleron B., Callard P., De Rycke Y., Carette M.F., Rosenwald J.C., Cosset J.M., Housset M., Touboul E. (2000). Evaluation of microscopic tumor extension in non–small-cell lung cancer for three-dimensional conformal radiotherapy planning.. Int. J. Radiat. Oncol. Biol. Phys..

[20] Zhang X., Zhang G., Qiu X., Yin J., Tan W., Yin X., Yang H., Liao L., Wang H., Zhang Y. (2023). Radiomics under 2D regions, 3D regions, and peritumoral regions reveal tumor heterogeneity in non-small cell lung cancer: A multicenter study.. Radiol. Med..

[21] Liu K., Li K., Wu T., Liang M., Zhong Y., Yu X., Li X., Xie C., Zhang L., Liu X. (2022). Improving the accuracy of prognosis for clinical stage I solid lung adenocarcinoma by radiomics models covering tumor per se and peritumoral changes on CT.. Eur. Radiol..

[22] Yang W., Li Z., Wang W., Wu J., Li J., Huang X., Zhang X., Ye X. (2023). Vasculogenic mimicry score identifies the prognosis and immune landscape of lung adenocarcinoma.. Front. Genet..

[23] Cheng Y., Xu S., Wang H., Wang X., Niu S., Luo Y., Zhao N. (2022). Intra- and peri-tumoral radiomics for predicting the sentinel lymph node metastasis in breast cancer based on preoperative mammography and MRI.. Front. Oncol..

[24] Jiang W., Meng R., Cheng Y., Wang H., Han T., Qu N., Yu T., Hou Y., Xu S. (2024). Intra‐ and peritumoral based radiomics for assessment of lymphovascular invasion in invasive breast cancer.. J. Magn. Reson. Imaging.

[25] Li S., Yang Z., Li Y., Zhao N., Yang Y., Zhang S., Jiang M., Wang J., Sun H., Xie Z. (2024). Preoperative prediction of vasculogenic mimicry in lung adenocarcinoma using a CT radiomics model.. Clin. Radiol..

[26] Shinohara S., Takahashi Y., Komuro H., Matsui T., Sugita Y., Demachi-Okamura A., Muraoka D., Takahara H., Nakada T., Sakakura N., Masago K., Miyai M., Nishida R., Shomura S., Shigematsu Y., Hatooka S., Sasano H., Watanabe F., Adachi K., Fujinaga K., Kaneda S., Takao M., Ohtsuka T., Yamaguchi R., Kuroda H., Matsushita H. (2022). New evaluation of the tumor immune microenvironment of non-small cell lung cancer and its association with prognosis.. J. Immunother. Cancer.

[27] Aldrees R., Siegal G.P., Wei S. (2023). The peritumoral CD8+/FOXP3+ cell ratio has prognostic value in triple-negative breast cancer.. Appl. Immunohistochem. Mol. Morphol..

[28] Lim J.U., Lee E., Lee S.Y., Cho H.J., Ahn D.H., Hwang Y., Choi J.Y., Yeo C.D., Park C.K., Kim S.J. (2023). Current literature review on the tumor immune micro-environment, its heterogeneity and future perspectives in treatment of advanced non-small cell lung cancer.. Transl. Lung Cancer Res..

[29] Shiinoki T., Fujimoto K., Kawazoe Y., Yuasa Y., Kajima M., Manabe Y., Ono T., Hirano T., Matsunaga K., Tanaka H. (2022). Predicting programmed death-ligand 1 expression level in non-small cell lung cancer using a combination of peritumoral and intratumoral radiomics features on computed tomography.. Biomed. Phys. Eng. Express.

[30] Chang R., Qi S., Zuo Y., Yue Y., Zhang X., Guan Y., Qian W. (2022). Predicting chemotherapy response in non-small-cell lung cancer *via* computed tomography radiomics features: Peritumoral, intratumoral, or combined?. Front. Oncol..

[31] Wang J., Xia W., Huang Y., Li H., Tang Y., Li Y., Yi B., Zhang Z., Yang J., Cao Z., Zhou J. (2022). A vasculogenic mimicry prognostic signature associated with immune signature in human gastric cancer.. Front. Immunol..

[32] Lapkina E.Z., Esimbekova A.R., Ruksha T.G. (2023). Vasculogenic mimicry.. Arkh. Patol..

[33] Tunali I., Hall L.O., Napel S., Cherezov D., Guvenis A., Gillies R.J., Schabath M.B. (2019). Stability and reproducibility of computed tomography radiomics features extracted from peritumoral regions of lung cancer lesions.. Med. Phys..

[34] Nayak P., Sinha S., Goda J.S., Sahu A., Joshi K., Choudhary O.R., Mhatre R., Mummudi N., Agarwal J.P. (2023). Computerized tomography-based first order tumor texture features in non-small cell lung carcinoma treated with concurrent chemoradiation: A simplistic and potential surrogate imaging marker for survival.. J. Cancer Res. Ther..

[35] Xu H., Wang A., Zhang C., Ren J., Zhou P., Liu J. (2023). Intra- and peritumoral MRI radiomics assisted in predicting radiochemotherapy response in metastatic cervical lymph nodes of nasopharyngeal cancer.. BMC Med. Imaging.

[36] Ganeshan B., Goh V., Mandeville H.C., Ng Q.S., Hoskin P.J., Miles K.A. (2013). Non-small cell lung cancer: Histopathologic correlates for texture parameters at CT.. Radiology.

[37] Provance O.K., Oria V.O., Tran T.T., Caulfield J.I., Zito C.R., Aguirre-Ducler A., Schalper K.A., Kluger H.M., Jilaveanu L.B. (2024). Vascular mimicry as a facilitator of melanoma brain metastasis.. Cell. Mol. Life Sci..

[38] Wang M., Zhao X., Zhu D., Liu T., Liang X., Liu F., Zhang Y., Dong X., Sun B. (2017). HIF-1α promoted vasculogenic mimicry formation in hepatocellular carcinoma through LOXL2 up-regulation in hypoxic tumor microenvironment.. J. Exp. Clin. Cancer Res..

[39] Shintani Y., Kimura T., Funaki S., Ose N., Kanou T., Fukui E. (2023). Therapeutic targeting of cancer-associated fibroblasts in the non-small cell lung cancer tumor microenvironment.. Cancers.

[40] Treps L., Faure S., Clere N. (2021). Vasculogenic mimicry, a complex and devious process favoring tumorigenesis – Interest in making it a therapeutic target.. Pharmacol. Ther..

