# Supplementary material for: Preoperative CT-based Intratumoral and Peritumoral Radiomics Prediction for Vasculogenic Mimicry in Lung Adenocarcinoma
Source: Curr Med Imaging. 2025 Apr 11;21:e15734056383032. doi: 10.2174/0115734056383032250320041531 (PMC13107382; doi:10.2174/0115734056383032250320041531)
Supplement: Supplementary file 1 [file CMIM-21-E15734056383032_SD1.pdf]

1573-4056/25

Send Orders for Reprints to reprints@benthamscience.net

BENTHAM SCIENCE

CrossMark

Current Medical Imaging

Content list available at: https://benthamscience.com/journals/cmir

Supplementary Material

Preoperative CT-based Intratumoral and Peritumoral Radiomics Prediction for Vasculogenic Mimicry in Lung Adenocarcinoma

Shuhua Li[1,2,3,#], Yang Li[4,#], Ying Meng[1], Jingcheng Huang[1], Yihong Gu[1], Yan Song[5], Shuni Zhang[1], Zhiya Zhang[1], Weiming Zhao[1] and Zongyu Xie[1,2,3,*]

[1]*Department of Radiology, The First Affiliated Hospital of Bengbu Medical University, Bengbu233004, China*
[2]*Department of Medical Imaging Diagnostics, Bengbu Medical University, Bengbu 233030, China*
[3]*Anhui Province Key Laboratory of Respiratory Tumor and Infectious Disease, Bengbu 233004, China*
[4]*Department of Radiology, Hospital of Stomatology Affiliated to Anhui Medical University, Hefei 230032, China*
[5]*Department of Radiology, Jieshou City People's Hospital, Fuyang 236500, China*

Appendix 1.

Feature Normalization Processing. We implemented a maximum absolute normalization technique to mitigate the impact of varying magnitudes in radiomics feature values on feature selection and model construction. This method ensures that all feature values are scaled to a standard range, thereby facilitating a more equitable contribution of each feature to the analytical process. The normalization procedure is detailed as follows:

$$f(x) = \frac{x}{|x|_{max}}$$

Where *x* and *f(x)* are the values of features before and after normalization, respectively After normalization, all features were transformed to between (-1, 1).

Appendix 2.

Radiomics Feature Extraction. Optimal feature screening (number)

By statistical methods, the important features for classification are screened out. The built-in evaluation criterion is f_classif. Compute the ANOVA F-value for the provided sample.

f_classif (X, y)

Parameters: X {array-like, sparse matrix} of shape (n_samples, n_features)

The set of regressors that will be tested sequentially.

y: ndarray of shape (n_samples,)

The target vector.

Returns: f_statisticndarray of shape (n_features,)

F-statistic for each feature.

p_valuesndarray of shape (n_features,)

P-values associated with the F-statistic.

Appendix 3.

Rad-Score of VOI - T = -1.252*lbp-3D-k_ngtdm_Contrast_HRCT_paramsName1+1.147*squareroot_glcm_ClusterShade_HRCT_paramsName1-1.147*wavelet-HHL_glcm_Correlation_HRCT_paramsName1-1.144*gradient_glcm_InverseVariance_HRCT_paramsName1-0.464*lbp-3D-k_firstorder_Kurtosis_HRCT_paramsName1-0.058*log-sigma-3-0-mm-3D_firstorder_TotalEnergy_HRCT_paramsName1+1.977

Rad-Score of VOI - P3 = -1.109*exponential_gldm_DependenceNonUniformity_HRCT_paramsName2-0.957*log-sigma-3-0-mm-3D_firstorder_Energy_HRCT_paramsName2-0.957*log-sigma-3-0-mm-3D_firstorder_TotalEnergy_HRCT_paramsName2-0.725*logarithm_glrlm_GrayLevelNonUniformity_HRCT_paramsName2+1.547

Rad-Score of VOI - P5 = +1.593*wavelet-LHH_firstorder_Skewness_HRCT_paramsName3-1.061*wavelet-LHH_glcm_Correlation_HRCT_paramsName3-0.950*logarithm_ngtdm_Contrast_HRCT_paramsName3-0.716*log-sigma-3-0-

DOI: 10.2174/0115734056383032250320041531, 2025, *21*, e15734056383032

mm-3D_glszm_SizeZoneNonUniformity_HRCT_paramsName3-0.256*log-sigma-3-0-mm-3D_glszm_GrayLevelNonUniformity_HRCT_paramsName3+1.125

Rad-Score of VOI - P7 = -1.911*wavelet-HHL_glcm_Correlation_HRCT_paramsName4-1.247*wavelet-HLL_firstorder_Mean_HRCT_paramsName4-1.236*log-sigma-3-0-mm-3D_firstorder_Median_HRCT_paramsName4-0.763*gradient_glszm_SizeZoneNonUniformityNormalized_HRCT_paramsName4+1.937

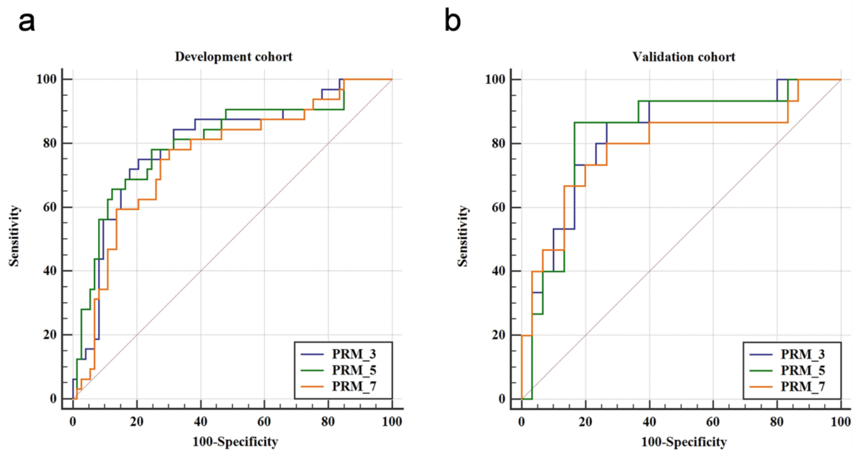

Fig. (1). ROC curves of PRM_3, PRM_5, and PRM_7 in the development (a) and validation (b) cohorts.PRM_3, 3-mm peritumoral radiomics model; PRM_5, 5-mm peritumoral radiomics model; PRM_7, 7-mm peritumoral radiomics model. ROC, receiver operating characteristic.

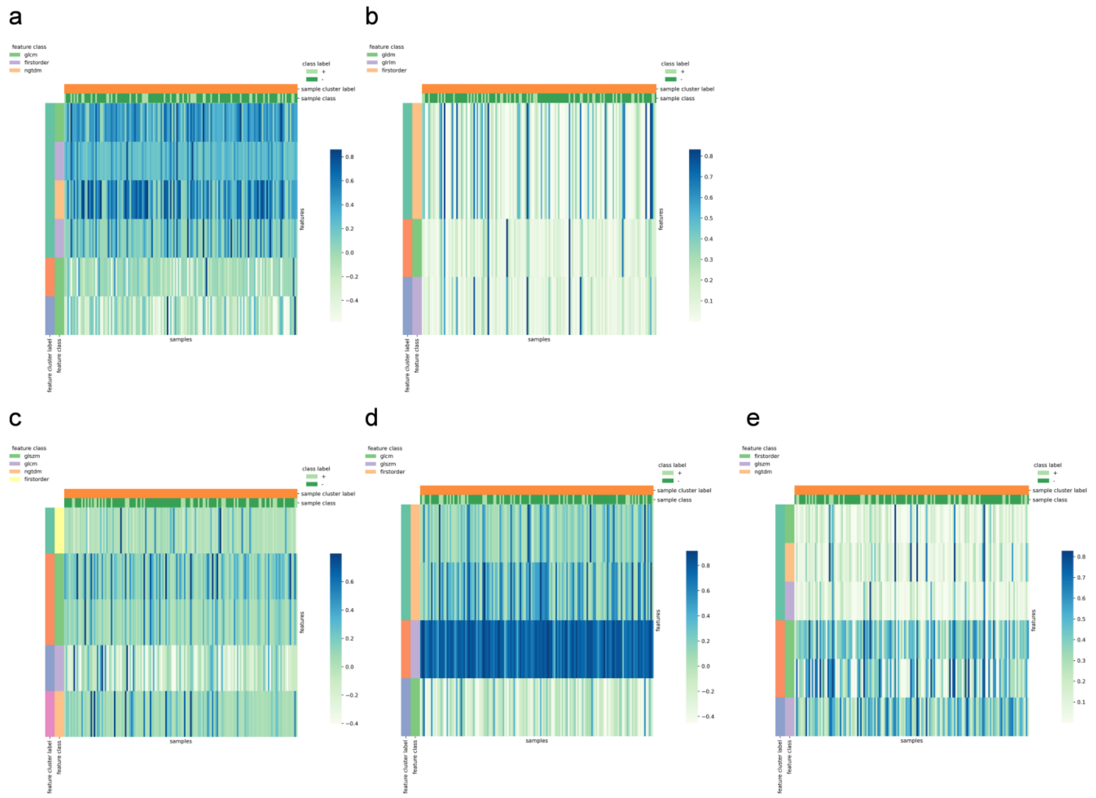

Fig. (2). Radiomics heatmap with 105 patients in the development cohort in the x-axis and 6, 4, 5, 4, and 6 optimal radiomics features from VOI-T(a), VOI-P3(b), VOI-P5(c), VOI-P7(d) and VOI-T+P5 (e) in the y-axis. K-means clustering revealed 2 clusters of patients with similar radiomics patterns in these models, and the VM status of LUAD was significantly different among these clusters. LUAD, lung adenocarcinoma; VM, vasculogenic mimicry; VOI-P3, 3 mm peritumoral volumes of interest; VOI-P5, 5 mm peritumoral volumes of interest; VOI-P7, 7 mm peritumoral volumes of interest; VOI -T, tumor volume of interest; VOI-T+P5, tumor and 7 mm peritumoral volumes of interest.

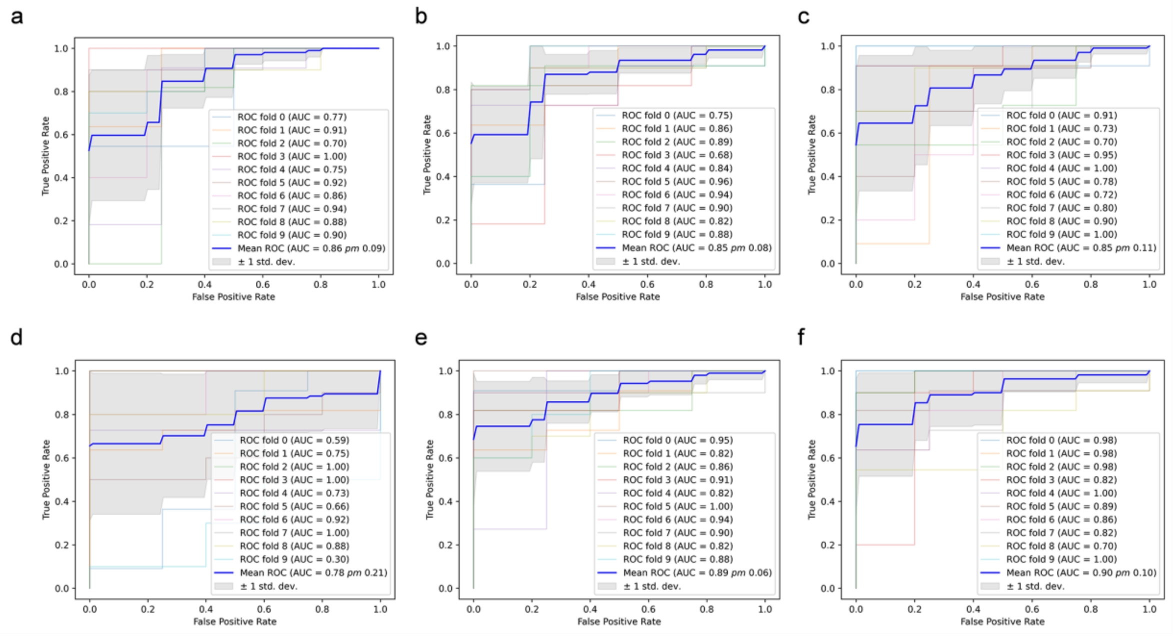

Fig. (3). 10-fold cross-validation of TRM(a), PRM_3(b), PRM_5(c), PRM_7(d), CRM(e), RNM(f). The blue line represents the average result of ten cross-validations. The gray shaded area is the corresponding standard deviation. CRM, combined intra- and peritumoral radiomics model; PRM_3, 3-mm peritumoral radiomics model; PRM_5, 5-mm peritumoral radiomics model; PRM_7, 7-mm peritumoral radiomics model; RNM, radiomics nomogram model; TRM, tumoral radiomics model.

Table 1. Top 6, 4, 5, 4, 6 critical features from VOI -T, VOI-P3, VOI-P5, VOI-P7 and VOI-T+P5.

VOI	Feature Group	Feature name	Feature_scores
VOI-T	NGTDM	lbp-3D-k_ngtdm_Contrast	1.252
	GLCM	squareroot_glcm_ClusterShade	1.147
		wavelet-HHL_glcm_Correlation	1.147
		gradient_glcm_InverseVariance	1.144
	first-order	3D-k_firstorder_Kurtosis	0.464
		log-sigma-3-0-mm-3D_firstorder_TotalEnergy	0.005
VOI-P3	GLDM	exponential_gldm_DependenceNonUniformity	1.109
	GLRLM	logarithm_glrlm_GrayLevelNonUniformity	0.725
	first-order	log-sigma-3-0-mm-3D_firstorder_Energy	0.957
		log-sigma-3-0-mm-3D_firstorder_TotalEnergy	0.957
VOI-P5	GLCM	wavelet-LHH_glcm_Correlation	1.593
	NGTDM	logarithm_ngtdm_Contrast	0.950
	GLSZM	log-sigma-3-0-mm-3D_glszm_SizeZoneNonUniformity	0.716
		og-sigma-3-0-mm-3D_glszm_GrayLevelNonUniformity	0.256
	first-order	wavelet-LHH_firstorder_Skewness	1.593
VOI-P7	GLCM	wavelet-HHL_glcm_Correlation	1.911
	GLSZM	lgradient_glszm_SizeZoneNonUniformityNormalized	0.763
	first-order	wavelet-HLL_firstorder_Mean	1.247
		log-sigma-3-0-mm-3D_firstorder_Median	1.236
VOI-T+P5	GLSZM	lbp-3D-k_glszm_ZonePercentage	1.084
		log-sigma-3-0-mm-3D_glszm_SizeZoneNonUniformity	0.559
	NGTDM	logarithm_ngtdm_Contrast	0.666
	first-order	log-sigma-3-0-mm-3D_firstorder_Maximum	0.808
		log-sigma-3-0-mm-3D_firstorder_Median	0.761
		logarithm_firstorder_RobustMeanAbsoluteDeviation	0.399

GLCM, gray level co-occurrence matrix; NGTDM, neighboring gray-tone difference matrix; GLDM, gray level dependence matrix; GLRLM, gray level run length matrix; GLSZM, gray level size zone matrix; VOI -T, tumor volume of interest; VOI-P3, 3 mm peritumoral volumes of interest; VOI-P5, 5 mm peritumoral volumes of interest; VOI-P7, 7 mm peritumoral volumes of interest; VOI -T+P5, tumor and 5 mm peritumoral volumes of interest.

Table 2. Uni- and multivariate logistic regression analysis of VM expression in LUAD in development cohort.

Characteristics		Univariate analysis		Multivariate analysis	
		OR (95%CI)	*P*	OR (95%CI)	*P*
Age		1.020 (0.983-1.059)	0.297		
Sex	Male	1			
	Female	0.769 (0.328-1.802)	0.545		
Size		5.262 (2.606-10.624)	0.000	3.606 (1.505-8.640)	0.004
Shape	Regular	1	0.062		
	Irregular	0.401 (0.154-1.047)			
Boundary	Clear	1	0.005	0.279 (0.061-1.275)	0.100
	Blur	0.281 (0.116-0.682)			
Lobulation	Absence	1	0.103		
	Present	0.379 (0.118-1.216)			
Spiculation	Absence	1	0.000	0.131 (0.040-0.428)	0.001
	Present	0.079 (0.029-0.213)			
Density	GGO or mGGO	1	0.003	1.316 (0.301-5.749)	0.715
	Consolidation	0.246 (0.098-0.620)			
Halo sign	Absence	1	0.029	1.848 (0.590-5.792)	0.292
	Present	2.667 (1.108-6.417)			
Calcification	Absence	1	0.041	0.102 (0.005-2.060)	0.137
	Present	0.097 (0.010-0.908)			
Necrosis	Absence	1	0.297		
	Present	0.414 (0.079-2.174)			
Vocule sign	Absence	1	0.167		
	Present	2.291 (0.707-7.419)			
Cavity	Absence	1	0.461		
	Present	2.297 (0.255-20.339)			
Air bronchogram	Absence	1	0.209		
	Present	1.860 (0.707-4.895)			
Peripheral fibrosis	Absence	1	0.091		
	Present	0.433 (0.164-1.142)			
Pleural retraction	Absence	1	0.052		
	Present	0.419 (0.174-1.007)			
Lymphadenectasis	< 10mm	1	0.727		
	≥ 10mm	1.343 (0.256-7.045)			

CI, confidence interval; GGO, ground-glass opacity; LUAD, lung adenocarcinoma; mGGO, mixed ground-glass opacity; OR, odds ratio; VM, vasculogenic mimicry. $P < 0.05$ is considered statistically significant.

© 2025 The Author(s). Published by Bentham Science Publisher.

This is an open access article distributed under the terms of the Creative Commons Attribution 4.0 International Public License (CC-BY 4.0), a copy of which is available at: https://creativecommons.org/licenses/by/4.0/legalcode. This license permits unrestricted use, distribution, and reproduction in any medium, provided the original author and source are credited.
